# Investigating Single Nucleotide Polymorphisms in the Etiology of Cleft Lip and Cleft Palate in the Polish Population

**DOI:** 10.3390/ijms25179310

**Published:** 2024-08-28

**Authors:** Alicja Zawiślak, Krzysztof Woźniak, Beata Kawala, Satish Gupta, Anna Znamirowska-Bajowska, Katarzyna Grocholewicz, Jan Lubiński, Anna Jakubowska

**Affiliations:** 1 Department of Maxillofacial Orthopaedics and Orthodontics, Institute of Mother and Child, 01–211 Warsaw, Poland; 2 Department of Interdisciplinary Dentistry, Pomeranian Medical University, 70–111 Szczecin, Poland; katarzyna.grocholewicz@pum.edu.pl; 3 Department of Orthodontics, Pomeranian Medical University, 70–111 Szczecin, Poland; krzysztof.wozniak@pum.edu.pl; 4 Department of Dentofacial Orthopaedics and Orthodontics, Wrocław Medical University, 50–425 Wrocław, Poland; ortodoncja@umed.wroc.pl (B.K.); aznamirowska.b@gmail.com (A.Z.-B.); 5 Hereditary Cancer Center, Department of Genetics and Pathology, Pomeranian Medical University, 70–111 Szczecin, Poland; satish1482@gmail.com (S.G.); jan.lubinski@pum.edu.pl (J.L.); anna.jakubowska@pum.edu.pl (A.J.); 6 Independent Laboratory of Molecular Biology and Genetic Diagnostics, Pomeranian Medical University, 70–111 Szczecin, Poland

**Keywords:** birth defect, congenital malformation, cleft lip, cleft palate, genetic variant, single nucleotide polymorphism

## Abstract

Cleft lip and/or palate (CL/P) are the most common congenital anomalies in the craniofacial region, leading to morphological and functional disruptions in the facial region. Their etiology involves genetic and environmental factors, with genetics playing a crucial role. This study aimed to investigate the association of four single nucleotide polymorphisms (SNPs)—rs987525, rs590223, rs522616, and rs4714384—with CL/P in the Polish population. We analyzed DNA samples from 209 individuals with CL/P and 418 healthy controls. The impact of SNPs on the presence of CL/P was assessed using multivariate logistic regression. Significant associations were found with rs987525. Specifically, the AC genotype was linked to an increased CL/P risk (odds ratio [OR] = 1.95, 95% confidence interval [CI]: 1.34–2.83, *p* < 0.001), while the CC genotype was associated with a decreased risk (OR = 0.46, 95% CI: 0.32–0.67, *p* < 0.001). Rs4714384 was also significant, with the CT genotype correlated with a reduced risk of CL/P (OR = 0.66, 95% CI: 0.46–0.94, *p* = 0.011). SNPs rs590223 and rs522616 did not show statistically significant associations. These results underscore the role of rs987525 and rs4714384 in influencing CL/P risk and suggest the utility of genetic screening in understanding CL/P etiology.

## 1. Introduction

The development of the cranial and facial parts of the skull is one of the most complex processes occurring during early embryogenesis. It requires the coordination of numerous factors that must work together in a precise manner. This intricate process involves the interaction of genetic, molecular, and even environmental factors to ensure proper formation of facial structures and the palate. Disruptions or malfunctions in any of these elements can lead to a range of developmental malformations. If developmental disruptions occur between the 4th and 12th weeks of fetal development, it can result in the formation of an orofacial cleft (OFC) [1].

There are various types of OFCs, including cleft lip (CL), cleft palate only (CPO), unilateral cleft lip and palate (UCL/P), and bilateral cleft lip and palate (BCL/P), each with a distinct possible etiology and characteristics. The development of OFCs is based on genetic predispositions and influenced by environmental factors. These conditions are the most common craniofacial birth defects, and they occur worldwide at rate of approximately 0.45 per 1000 live newborns [2]. A cleft defect significantly impacts a child’s development in multiple ways. It not only negatively affects the function of the stomatognathic system, impairing essential functions such as eating, speaking, and breathing, but it also has profound psychological and socioeconomic repercussions. Children with birth defects often face challenges related to self-esteem and social integration, while their families may encounter financial and emotional strain due to the lifelong medical care required [3]. Treatment for OFCs is complicated, involves a multidisciplinary approach, and requires a team of specialists to address the complex needs of patients with OFCs. This team typically includes plastic surgeons, orthodontists, otolaryngologists, speech therapists, and geneticists [4].

Single nucleotide polymorphisms (SNPs) play a significant role in the development of cleft lip and palate (CL/P). These genetic variations influence an individual’s susceptibility. Research has identified several SNPs associated with an increased risk of OFC, highlighting the importance of genetic screening and personalized medical approaches. Studying SNPs helps researchers to identify potential targets for future therapeutic interventions and improve treatment strategies for affected patients [5]. Moreover, the specific SNPs associated with OFCs vary across different populations. They may be more prevalent or have a greater impact in one population compared to another, so conducting population-specific genetic researches is crucial [6].

The available literature and research, particularly previous genome-wide association studies (GWAS), suggest that specific SNPs may correlate with CL/P in other populations. It is reasonable to select a smaller subset of SNPs for focused investigation. Examining these SNPs within a homogenous population can provide more precise insights into their potential role in the development of CL/P. This approach not only builds on the findings of earlier research but also aims to enhance our understanding of the genetic factors contributing to these conditions in a specific demographic context [7,8].

Numerous studies have suggested that certain gene variants are possibly strongly associated with CL/P occurrence across different populations. Among them rs987525, rs590223, rs522616, and rs4714384 have been highlighted due to their roles in critical genetic pathways related to craniofacial development. Investigating these SNPs can provide valuable insights into their contribution to the risk of CL/P, helping to unravel the complex genetics of this malformation and leading to better diagnostic and therapeutic strategies [9,10,11,12]. The SNP rs987525 has consistently shown strong associations with CL/P across various populations. For instance, rs987525 was significantly associated with CL/P in an Italian cohort, with a homozygous relative risk of 3.60 (95% CI, 2.12–6.13). This finding aligns with numerous research studies demonstrating similar associations in diverse populations, highlighting rs987525 as a robust genetic marker for CL/P [13].

Examining these SNPs to determine whether they are associated with an increased or reduced risk of CL/P, and to further our understanding of the genetic basis of these congenital malformations, seems to be of greater importance. This approach might allow us to explore genetic factors that may contribute to the etiology of CL/P, potentially leading to improved risk prediction and targeted interventions. This study of the association between OFCs and SNPs holds significant potential to benefit clinicians in addressing the challenges posed by these congenital anomalies. By identifying genetic predispositions and understanding the role of specific SNPs, clinicians can better predict the risk of OFCs, especially in families with a history of such conditions. This knowledge aids in early diagnosis and intervention and facilitates the development of personalized treatment plans. Furthermore, insights into the genetic and environmental interactions contributing to OFCs can inform targeted prevention strategies, ultimately reducing the incidence of these conditions.

The aim of our study was to investigate the correlations between SNPs rs987525, rs590223, rs522616, and rs4714384 and their potential influence on the occurrence of CL/P in the Polish population.

## 2. Results

Our study involved a total of 627 patients. They were divided into two groups: 209 individuals diagnosed with OFC and 418 healthy individuals in the control group, randomly selected from orthodontic patients. The ages of individuals ranged from 4 to 30 years, with a mean age of 17.4 ± 13.6 years, and a median age of 16.8 years. The groups had identical gender distributions. Females comprised 43.54% of each group: 91 in the study group, and 182 in the control group. Males comprised 56.46%: 118 in the study group, and 236 in the control group. Individuals in the study group were divided into four categories based on type of cleft. Most patients were diagnosed with UCL/P (54%). 21% were diagnosed with BCL/P, 15% with CPO. Only 9% of the individuals were diagnosed with CL. This distribution underscores the variety of OFC within the cohort.

When examining SNPs, different genotype distributions were observed between the OFC and control group for the studied SNPs. This data is summarized in Table 1.

We have conducted a comprehensive evaluation of the possible associations between different genetic variants and the occurrence of OFC, incorporating sex as a confounding variable. The results are presented in Table 2. This adjustment allows for a more accurate evaluation by considering confounding factors across various strata, thereby improving the reliability of clinical assessments. Such a comprehensive analysis deepens our understanding of the complex genetic vulnerabilities linked to OFC, aiding in the creation of targeted genetic screening protocols and clarifying the role of sexual dimorphism in these susceptibilities.

Since the stratum-specific OR in Table 2 do not show significant differences, the Mantel–Haenszel adjusted OR is an appropriate summary measure to explain the link between genotypes and OFC occurrence. Including specific genotypes with significant associations in genetic screening could greatly improve our understanding and management of birth defects, allowing for earlier interventions.

Exhibiting an OR = 1.95, carriers of rs987525 AC genotype fall into the high-risk category of patients. These individuals should undergo prenatal monitoring more often and engage early with pediatric craniofacial specialists.

With an OR = 0.46 and OR = 0.66 respectively, indicating a lower risk of OFC, individuals with the rs987525 CC and rs4714384 CT genotypes might not require the intensive surveillance protocols meant for higher-risk genotypes.

The effect of genotypes on OFC incidence was consistent across sexes, showing a homogeneous impact. This suggests a stable genetic influence on the risk of OFC occurrence, regardless of sex, which supports using these genotypes in risk models without sex differentiation.

The analysis of the designated genotypes used multivariate models, with results as shown in Table 3. Rs987525 AC, and rs987525 CC remained statistically significant even after excluding incomplete data, indicating a strong link to OFC occurrence and reinforcing their role as important genetic markers. Due to multicollinearity, rs987525 genotypes were evaluated independently (model 1 for rs987525 AC and model 2 for rs987525 CC) to maintain statistical accuracy.

On the other hand, rs4714384 CT showed a trend toward association (*p* < 0.200) but did not reach significance levels. Although not conclusively linked to OFC in our study, this genotype may still be clinically relevant and require further investigation in larger studies to determine its effects.

The strength of the association between genotypes and the occurrence of specific cleft types was assessed. Table 4 exclusively lists the genotypes that have demonstrated significant associations.

According to the data in the table above, two genotypes showed significant associations. Of these, rs522616 AA and rs987525 CC were identified as having protective effects against certain OFCs. In contrast, rs987525 AC was associated with an increased risk of developing OFC. Sex does not significantly influence the link between genotype and the occurrence of specific cleft types. This supports the notion that it is primarily genetic factors that drive the development of these conditions, regardless of sex differences. The multivariate analysis results in Table 5 reveal that rs522616 AA, rs987525 AC, and rs987525 CC maintained their significance within the multivariate framework. This consistency highlights the strong associations of these genotypes and their potential importance in the genetic etiology of OFC. For CL, the findings were less conclusive, particularly within the multivariate framework, due to the malformation’s relative rarity. This underscores the problem of achieving strong statistical power and emphasizes the need for extensive research. Moreover, this genotype has shown notable effects in multivariable analyses, reinforcing its potential as an essential genetic marker for OFCs.

## 3. Discussion

Our findings reinforce the well-documented influence of specific SNPs on the occurrence of craniofacial birth defects in the Polish population. Notably, genotype rs987525 has been shown to either lower or increase the risk of OFCs among newborns. Located on chromosome 8q24.21, rs987525 has been previously identified as significantly associated with OFCs at a genome-wide level, with an association reported at *p* = 9.18 × 10^−8^ and an odds ratio of 2.09 (95% CI = 1.59 to 2.76). This association was replicated in a genome-wide association study of OFC in a German cohort, confirming the role of this genetic marker in the risk of OFCs and supporting its possible relevance across different populations [14]. A similarly designed study involving a genome-wide association study with 224 cases and 383 controls of Central European origin identified key susceptibility loci for OFCs. The study pinpointed the 640-kb region 8q24.21, which harbored markers with highly significant associations to OFC. Within this region, the SNP rs987525 was found to be the most significant. In the expanded analysis of 462 OFC cases and 954 controls, rs987525 displayed an exceptionally low *p*-value of 3.34 × 10^−24^. The ORs for rs987525 were substantial: 2.57 (95% CI = 2.02–3.26) for the heterozygous genotype and 6.05 (95% CI = 3.88–9.43) for the homozygous genotype. The population’s attributable risk for rs987525 was estimated at 0.41, highlighting its importance as a major genetic predisposition to this condition [15].

The results from the studies of rs987525 in different populations show remarkable consistency. In the Central European cohort, German cohort, and our Polish study, rs987525 demonstrated a strong association with non-syndromic OFC. These similar findings across diverse groups reinforce the SNP’s relevance and potential utility in understanding and managing the genetic risk of this condition. Our results are compatible with research conducted by Mostowska A. et al. They examined 18 polymorphisms in the Polish population, and the SNP rs987525 in the 8q24 region demonstrated a strong association with CL/P occurrence. Rs987525 showed a significant OR of 1.962 (95% CI = 1.382–2.785; *p* = 1.4 × 10^−4^, indicating a notably increased risk of OFC. This association remained significant even after correcting for multiple comparisons, underscoring the importance of rs987525 in the genetic susceptibility to clefts in the Polish population and is in accordance with our findings [16]. Moreover, studies on a closely related ethnic population have also demonstrated the impact of this polymorphism on the development of OFCs, suggesting that rs987525 may contribute to the risk of CL/P in the Slovak population. In that study, rs987525 was analyzed in 165 CL/P patients and 326 healthy controls using high-resolution melting analysis after real-time PCR. As a result, significant differences in allele and genotype frequencies between patient and control groups were found [17].

It is intriguing that rs987525 may not only be associated with OFC but also predispose individuals to cancers, as described by de Freitas E.M. et al. This dual role highlights the potential significance of rs987525 in various genetic and clinical contexts. The study evaluated the association of the SNP with oral and breast cancer in a Brazilian population. For breast cancer, the A allele of rs987525 was associated with an increased risk in the early stage (*p* = 0.02). These findings suggested that this SNP, known to be associated with OFC, may also be relevant in assessing cancer risk [18].

For SNP rs590223, no significant association with CL/P was detected in our study, which is similar to studies conducted by Brito L.A. et al. in the studied Brazilian population. Additionally, there was no correlation observed between the genotypes of rs590223 and expression levels in mesenchymal stem cells (MSC). These findings suggest that rs590223 may not have a detectable impact on the development of CL/P or on gene expression in the context studied. Its potential functional role, if any, might be related to specific periods of embryogenesis that were not assessed in this study [19]. Despite previous studies showing negative results, investigating this SNP could provide additional insights into its potential effects or interactions with other genetic factors.

The next polymorphism examined in our study was rs522616. Although our preliminary analysis did not find a significant association, we included rs522616 as an important possible risk factor, based on evidence from other studies that have identified its potential role in CL/P. Statistical analysis did not confirm a significant impact of this genetic variant on the risk of CL/P. However, different conclusions were reached by Letra et al. [20], who studied 494 individuals with CL/P and 413 healthy controls. They found that the rs522616 polymorphism in the *MM3* gene was associated with the occurrence of clefts in the Brazilian population. This study is unique in focusing on the association between this polymorphism and clefts. Additionally, the literature includes publications describing the co-occurrence of rs522616 with ovarian cancer [21], gastric cancer [22], and coronary artery disease [23].

Similarly, rs4714384 was found as not associated with a modified risk of CL/P in our study, but as has been proven by other researchers, this polymorphism is likely a significant biomarker for cardiovascular events in end-stage renal disease patients. This suggests that genetic variants in the *EDN1* gene could play a critical role in assessing cardiovascular risk in this high-risk population [24].

An important aspect to consider is the potential role of epigenetic factors, which may influence their expression. Emerging evidence suggests that environmental factors such as diet, nutrition, physical activity, and exposure to chemicals during pregnancy can affect epigenetic modifications, potentially altering the risk and severity of CL/P. For instance, maternal folate levels and exposure to teratogens have been shown to interact with genetic predispositions in CL/P development. Additionally, other potential factors, such as maternal age, smoking, and socioeconomic status, also merit consideration as they may contribute to the overall risk of CL/P. These factors, in combination with genetic predispositions, underscore the multifactorial nature of CL/P and highlight the need for a comprehensive approach to studying its etiology [25]. There are currently no specific literature data comparing these additional factors with the specific SNPs we studied.

The findings of this study have important implications for clinical practice, particularly in mitigating the challenges associated with OFCs. The identification of SNPs associated with these anomalies allows clinicians to assess the risk and offer more precise genetic counseling to at-risk families. This can lead to more informed decision-making and early interventions. Additionally, the ability to tailor treatment plans based on a patient’s genetic profile represents an advancement in personalized medicine. Clinicians can optimize therapeutic approaches, enhancing the effectiveness of interventions. Furthermore, recognizing the link between certain SNPs and environmental factors provides an opportunity to develop targeted prevention strategies, reducing the overall burden of OFCs in the population.

### Study Limitations

Research on OFCs has often produced inconsistent findings. We acknowledge that our study design, which examines the correlation between SNPs and various phenotypes, may have limited power to detect the complex inheritance patterns involved, such as the combined effects of multiple SNPs. Additional studies are necessary to validate the associations we reported. When assessing the association between single genes and the risk of complex traits like OFCs, ORs tend to be low to moderate, reflecting the reality that specific phenotypes emerge from the interplay of multiple genes (each with a small effect) and environmental factors. Therefore, it is crucial to explore gene–environment interactions to fully understand the remaining genetic risks associated with OFCs.

## 4. Conclusions

The study revealed a strong association between specific SNP genotypes and the incidence of cleft lip and/or palate (CL/P) in the Polish population. Notably, SNP rs987525 was identified as a significant genetic variant related to CL/P. Specifically, the AC genotype of rs987525 was associated with an increased risk of CL/P, whereas the CC genotype was linked to a decreased risk. Additionally, SNP rs4714384 showed significant findings, with the CT genotype associated with a reduced risk of CL/P. In contrast, the SNPs rs590223 and rs522616, as well as rest of genotypes of rs987525 and rs4714384, did not demonstrate statistically significant associations with CL/P in this population. The key takeaway from this study is the identification of rs987525 and rs4714384 as critical genetic markers influencing the CL/P risk in the Polish population. These findings emphasize the potential of these SNPs in genetic screening and early risk assessment, which could lead to more personalized approaches to managing CL/P.

## 5. Materials and Methods

### 5.1. Study Group

In our research, an unselected group of patients with CL/P (n = 209) was examined. The participants were undergoing orthodontic treatment at two Polish cleft centers: the Department of Orthodontics at Pomeranian Medical University in Szczecin and the Department of Dentofacial Orthopedics and Orthodontics at Wroclaw Medical University. The median age of participants was 16.8 years.

Inclusion criteria were as follows:

- Diagnosis of non-syndromic CL/P,
- Absence of other birth defects or genetic syndromes,
- Polish ancestry extending to two generations prior,
- Availability of comprehensive medical histories, anamneses, and clinical examination data.

Exclusion criteria were as follows:

- Presence of syndromic cleft lip and/or palate.
- Insufficient or incomplete medical history and clinical examination data.
- Severe systemic health conditions that could impact the study or treatment outcomes.

The study involved diagnosing existing congenital defects and identifying differential diagnoses for monogenic syndromes associated with CL/P. Detailed medical histories, anamneses, and clinical examinations were performed. Birth defects were categorized according to the World Health Organization’s International Statistical Classification of Diseases and Related Health Problems–ICD-10, focusing on sections Q35–Q37, which pertain to congenital malformations, deformations, and chromosomal abnormalities [26].

### 5.2. Control Group

The control group consisted of randomly selected newborns (n = 418), with their genetic material stored in the biobank of the Department of Genetics and Pathology at Pomeranian Medical University in Szczecin. Both groups were matched by age and geographical location to ensure comparability regarding environmental factors.

### 5.3. Ethical Approval

The study was approved by the Bioethics Committee of Pomeranian Medical University in Szczecin and adhered to Good Clinical Practice (GCP) standards (KB-0012/77/10). Additionally, the ethics committee approved the oncology biobank project (BN-001/174/05 dated 11 October 2005). All participants, or their legal guardians, provided detailed informed consent before participating in the study.

### 5.4. Genetic Variants Selection and Genotyping

Individuals in the study group with congenital defects provided 2 mL saliva samples using Oragene collection kits (DNA Genotek Inc., Ottawa, ON, Canada). Participants were instructed not to eat or drink for thirty minutes prior to saliva collection. The samples were kept at 22 °C in a dry and light-protected place. DNA isolation was performed with an automated Chemagen system, and the extracted DNA was stored at −20 °C. For the control group, DNA was extracted from umbilical cord blood using the standard procedure described by Lahiri et al. [27].

We selected four SNPs from four distinct genes previously proposed as potential variants linked to craniofacial birth defects and examined their association in the Polish population.

Although the samples were collected from saliva in one group and from umbilical cord blood in another, there are no significant differences between these sources that would affect the genotyping results. Both saliva and umbilical cord blood are reliable sources for DNA extraction, and standardized procedures were followed to ensure consistency and accuracy across all samples. Therefore, the variations in sample sources are not expected to influence the outcomes of the study.

The characteristics of the studied genetic variants are presented in Table 6.

Genotyping of selected gene variants (rs987525, rs590223, rs522616, and rs4714384) was carried out using the real-time PCR-based TaqMan method on the LightCycler 480 II (Roche Diagnostics, Basel, Switzerland). The reaction mixture for the PCR reaction and the PCR protocol followed the conditions were described in a previous study [28].

### 5.5. Statistical Analysis

Categorical variables were summarized by reporting counts (n) and percentages (%) for each category. The Mantel–Haenszel (M–H) chi-squared test, incorporating sex as a stratifying variable, was utilized to examine the association between specific SNP genotypes and the incidence of CL/P, controlling for potential confounders. This case–control study quantified the association by computing the odds of exposure, which were x times higher (or lower) in cases relative to controls.

The M–H method tested the null hypothesis that the combined OR is 1.0, using a two-sided test without continuity correction. The Wald confidence interval (CI) provided adjustments for the M–H odds ratio [29]. These M–H adjusted association measures are deemed robust and reliable when the association metrics across different stratifications are homogeneous, as indicated by a non-significant Woolf test for homogeneity of OR [30].

The influence of SNP genotypes on CL/P occurrence was assessed via multivariate analysis using multiple logistic regression. This model employed a binomial distribution and a logit link function, with parameters estimated by maximum likelihood. The precision of these estimates was evaluated through *p*-values and confidence intervals derived from the Wald z statistic. Multicollinearity within the regression model was evaluated using the Variance Inflation Factor (VIF), with values exceeding 3.0 suggesting significant multicollinearity.

Analyses were performed using the R statistical software [31] on a Windows 10 Pro 64-bit system (build 19045), utilizing the packages sjPlot [32], performance [33], report [34], gtsummary [35], epiR [36], and dplyr [37]. The statistical significance threshold was established at an alpha level of α = 0.05.

## Figures and Tables

**Table 1:** Genotype distributions of studied genetic variants in OFC and healthy patients.

Genetic Variant	*N* (total)	Genotype	Study Group *n* = 209	Control Group *n* = 418
rs987525		AA	13 (7.22%)	14 (4.06%)
	525	AC	79 (43.89%)	99 (28.70%)
		CC	88 (48.89%)	232 (67.25%)
rs590223		AA	76 (46.06%)	133 (45.24%)
	459	AG	71 (43.03%)	138 (46.94%)
		GG	18 (10.91%)	23 (7.82%)
rs522616	494	AA	125 (65.10%)	207 (68.54%)
		AG	61 (31.77%)	83 (27.48%)
		GG	6 (3.13%)	12 (3.97%)
rs4714384	552	CC	20 (10.47%)	30 (8.31%)
		CT	80 (41.88%)	188 (52.08%)
		TT	91 (47.64%)	143 (39.61%)

**Table 2:** Assessment of the strength of the relationship between genotypes and OFC incidence, adjusting for the influence of sex through stratified analysis.

Genetic Variant	Genotype	OR with CI 95%	p_M-H adj OR=1_	p_Woolf homogeneity_
rs987525	AA	1.86 (0.85–4.06)	0.057	0.962
rs987525	AC	1.95 (1.34–2.83)	<0.001	0.226
rs987525	CC	0.46 (0.32–0.67)	<0.001	0.296
rs590223	AA	1.04 (0.71–1.52)	0.428	0.391
rs590223	AG	0.85 (0.58–1.25)	0.209	0.388
rs590223	GG	1.44 (0.75–2.75)	0.137	0.899
rs522616	AA	0.86 (0.58–1.26)	0.213	0.694
rs522616	AG	1.23 (0.83–1.82)	0.151	0.647
rs522616	GG	0.77 (0.28–2.10)	0.307	0.869
rs4714384	CC	1.30 (0.72–2.36)	0.194	0.338
rs4714384	CT	0.66 (0.46–0.94)	0.011	0.573
rs4714384	TT	1.39 (0.97–1.98)	0.051	0.300

*Note: Incidence OR*—the outcome incidence odds ratio with a confidence interval of 95% among the exposed outcome incidence to those among the unexposed after accounting for confounding; *p_M-H adj OR=1_*—the *p*-value of test for equality of Mantel–Haenszel adjusted OR to 1; *p_Woolf homogeneity_*—the *p*-value of Woolf test homogeneity of the strata OR.

**Table 3:** The regression coefficients of the multiple logistic regression models with OFC incidence as the outcome variable, N_obs_ = 439.

Predictors	OFC					
	Model 1			Model 2		
	OR	CI 95%	*p*	OR	CI 95%	*p*
(Intercept)	0.41	0.27–0.59	<0.001	0.86	0.57–1.29	0.458
rs4714384 CT	0.72	0.47–1.09	0.123	0.72	0.47–1.09	0.119
rs987525 AC	1.95	1.28–2.98	0.002	-	-	-
rs987525 CC	-	-	-	0.43	0.28–0.65	<0.001

**Table 4:** Assessment of association strength (significant only) between genotypes and cleft types, adjusted for sex confounding.

CL/P	SNP	Genotype	OR with CI 95%	p_M-H adj OR=1_
BCL/P	rs522616	AA	0.53 (0.28–0.99)	0.022
CL	rs522616	AA	0.53 (0.28–0.99)	0.022
UCL/P	rs987525	AC	2.40 (1.54–3.73)	<0.001
	rs987525	CC	0.37 (0.24–0.58)	<0.001

**Table 5:** The regression coefficients of the multiple logistic regression model with BCL/P (N = 446), CL (N = 419), UCL/P (N = 411).

Predictor	OFC	OR	CI 95%	*p*
(Intercept)	BCL/P	0.11	0.05–0.19	<0.001
rs522616 AA	BCL/P	0.46	0.23–0.93	0.029
(Intercept)	CL	0.03	0.01–0.12	<0.001
rs522616 AA	CL	0.43	0.13–1.44	0.162
(Intercept)	UCL/P	0.12	0.05–0.19	<0.001
rs987525 AC	UCL/P	2.66	1.58–4.50	<0.001
(Intercept)	UCL/P	0.33	0.20–0.52	<0.001
rs987525 CC	UCL/P	0.31	0.18–0.52	<0.001

**Table 6:** Characteristics of studied SNPs.

SNP	Location	Position	Function	Alleles	MAF
rs987525	Chromosome 8q24.21	chr8:128933908	N/A	C > A	A = 0.0224
rs590223	TRAF3IP3	chr1:209773362	Intron Variant	G > A	G = 0.3666
rs522616	MMP3	chr11:102844317	2KB Upstream Variant	T > C	C = 0.2059
rs4714384	EDN1	chr6:12297620	500B Downstream Variant	T > C	C = 0.3304

## Data Availability

All data are available from the corresponding author upon resonable request.
